# Comparative transcriptome analysis between planarian *Dugesia japonica* and other platyhelminth species

**DOI:** 10.1186/1471-2164-13-289

**Published:** 2012-06-29

**Authors:** Osamu Nishimura, Yukako Hirao, Hiroshi Tarui, Kiyokazu Agata

**Affiliations:** 1 Department of Biophysics and Global COE Program, Graduate School of Science, Kyoto University, Kitashirakawa-Oiwake, Sakyo-ku, Kyoto, Japan; 2 Genome Resource and Analysis Unit, RIKEN Center for Developmental Biology, 2-2-3 Minatojima-Minamimachi, Chuo-ku, Kobe, Japan; 3 Functional Probe Research Laboratory, RIKEN Center for Molecular Imaging Science, 6-7-3 Minatojima-Minamimachi, Chuo-ku, Kobe, Japan; 4 LSA System Development Unit, RIKEN Omics Science Center, 1-7-22 Suehiro-cho, Tsurumi-ku, Yokohama, Japan

## Abstract

**Background:**

Planarians are considered to be among the extant animals close to one of the earliest groups of organisms that acquired a central nervous system (CNS) during evolution. Planarians have a bilobed brain with nine lateral branches from which a variety of external signals are projected into different portions of the main lobes. Various interneurons process different signals to regulate behavior and learning/memory. Furthermore, planarians have robust regenerative ability and are attracting attention as a new model organism for the study of regeneration. Here we conducted large-scale EST analysis of the head region of the planarian *Dugesia japonica* to construct a database of the head-region transcriptome, and then performed comparative analyses among related species.

**Results:**

A total of 54,752 high-quality EST reads were obtained from a head library of the planarian *Dugesia japonica*, and 13,167 unigene sequences were produced by *de novo* assembly. A new method devised here revealed that proteins related to metabolism and defense mechanisms have high flexibility of amino-acid substitutions within the planarian family. Eight-two CNS-development genes were found in the planarian (cf. *C. elegans* 3; chicken 129). Comparative analysis revealed that 91% of the planarian CNS-development genes could be mapped onto the schistosome genome, but one-third of these shared genes were not expressed in the schistosome.

**Conclusions:**

We constructed a database that is a useful resource for comparative planarian transcriptome studies. Analysis comparing homologous genes between two planarian species showed that the potential of genes is important for accumulation of amino-acid substitutions. The presence of many CNS-development genes in our database supports the notion that the planarian has a fundamental brain with regard to evolution and development at not only the morphological/functional, but also the genomic, level. In addition, our results indicate that the planarian CNS-development genes already existed before the divergence of planarians and schistosomes from their common ancestor.

## Background

The flatworm planarian *Dugesia japonica* inhabits fresh water in East Asian areas and is the most common planarian in Japan [1]. Planarians are members of the phylum Platyhelminthes, a group of organisms that are thought to have acquired a central nervous system (CNS) with a simple brain structure early during evolution [2-4]. The constitution of the planarian CNS and neural network has been visualized in detail by whole mount *in situ* hybridization and immunofluorescence studies, and fluorescent dye tracing experiments [5-8]: the CNS is composed of a brain (anterior cephalic ganglion) in the head region and a pair of ventral nerve cords extending anterior to posterior along the ventral side of the body, with the brain and ventral nerve cords being morphologically distinguishable structures. Neurons from a variety of sensory organs, such as eyes and auricles, project to different portions of the main lobes [6,8,9]. These interneurons are involved in information processing of external signals and work to regulate behavior and learning/memory [10-12]. The planarian brain acts as an information center of the nervous system, and shows fundamental evolutionarily conserved features of the animal brain not only morphologically but also functionally [12]. In addition, planarians are attracting attention as new model organism for regeneration, including brain regeneration, research. Interestingly, planarians can regenerate their well-organized brains from any portion of their bodies utilizing adult somatic pluripotent stem cells (neoblasts) [13-15]. The RNA interference technique can be applied in planarians to identify gene functions [16], and single-cell level gene profiling is also possible based on a combination of fluorescence activated cell sorting (FACS) and quantitative reverse transcription polymerase chain reaction (qRT-PCR) [17,18].

In recent years, resources for comparative transcriptome analysis among members of the phylum Platyhelminthes have accumulated rapidly. The planarian *Schmidtea mediterranea* and blood-fluke *Schistosoma mansoni* genome sequences have been analyzed [19,20], and transcriptome resources and analyses have been reported [21-23]. Unigene Build #4 for planarian *S. mediterranea,* which is based on the Sanger sequencing method, contains 10,173 clusters from 69,699 EST sequences, which were obtained from juvenile and adult libraries [24,25]. Some studies with massive numbers of sequencing reads produced from next generation sequencing technologies, include the Illumina HiSeq, Roche 454 and Life Technologies SOLiD, have been reported [26-28]. However, there is no genomic resource for *D. japonica*, and only limited transcriptome information is available for this species. Despite the large evolutionary distance between these two planarians [29], they share not just morphological similarity, but also genes, CNS features and regeneration ability [30].

The cDNA libraries of the schistosome *S. mansoni* cover its various life stages: egg, miracidium, sporocyst, cercaria, larva and adult, with a total of 152,704 sequences and 10,061 clusters (Unigene Build #19) [24]. Schistosomes are triploblastic animals and members of Platyhelminthes, like planarians, with which they share not only body shape but also basic organismal functions. Specifically, they have bilateral symmetry, a functional brain and peripheral nerves, ventral suckers, digestive and excretory organs; and lack a cardiovascular system [31]. Moreover, many genes and their amino acid sequences are well conserved between schistosomes and planarians. However, whereas planarians are free-living flatworms that prey on other organisms, schistosomes, which are major agents of the disease schistosomiasis and parasitize multiple hosts and organs, change their morphology to adapt to their living environments [32]. The life cycles of these two genuses are thus in sharp contrast, requiring brain functions and metabolic processes that are quite different.

To establish a database of genetic information for planarian transcriptome studies, we performed a large-scale EST project for the planarian *D. japonica* using head cDNA libraries. We adopted Sanger sequencing in order to decrease the sequence gaps, frame-shift errors, and the misassembly that can occur due to splice variants and to the short reads produced by next generation sequencing. These factors are important for the identification of long consensus sequences between conserved proteins. We compared the percentage of amino acid substitutions between *D. japonica* and its sister species *S. mediterranea* using the homologue proteins to identify genes whose mutability enables accommodation to different environmental conditions. For this analysis, we developed a method to extract gene groups that have different rates of evolution in close species that have very-well-conserved proteins.

We have already published a partial analysis of *D. japonica* transcriptome [33], and have identified several genes that are specifically expressed in the CNS [7,9,34]. However, those studies were insufficient for the exhaustive comparative analyses between planarians and members of the same family or the same phylum necessary for clarifying the composition and evolution of the CNS. As compared with model organisms, the gene information of Platyhelminthes is very limited. For these reasons we used Gene Ontology [35], which is based on information across many species, including vertebrates and non-vertebrates, and serves as a common platform to compare and annotate non-model organisms.

In this study, we focused on the CNS-development genes, which should give information about the evolutionary position of Platyhelminthes. To examine the genomic evolution and the presence of gene expression, we compared the *D. japonica* unigenes with not only *S. mansoni* unigenes but also the predicted protein information from the genome sequence. The traces we thereby found on the genome suggested the possibility that these genes were derived from the common ancestor of these two genuses, and the divergent gene expression between these genuses provided information about their adaptation to their specific habitats.

## Results

### EST sequencing

A non-normalized cDNA library was constructed using poly(A)+ RNA isolated from the heads of adult planarians. Two different experimental methods and DNA sequencers were used for the cDNA template amplification and DNA sequencing reaction (Table 1). After trimming of vector sequences, about 84.7% of reads passed the high-quality control for Phred base calling [36], and finally a total of 35,698 5’-end and 18,461 3’-end reads enabled the assembly analysis to proceed accurately. For 593 clones, the reading gap was closed to get the whole clone sequence by the primer walking method using custom primers based on the EST sequence. All EST reads and full-length clone sequences have been submitted to DDBJ. The accession numbers are 5’ ESTs [DDBJ: FY925127 - FY960824], 3’ ESTs [DDBJ: FY960825 - FY979285] and full-insert sequences [DDBJ: AK388576 - AK389168].

**Table 1:** Summary of the materials used in the EST analysis

**Library**	**Prefix**	**Plate number**	**cDNA amplification**	**Sequence primer**	**DNA sequencer**	**Valid 5' reads**	**Valid 3' reads**	**Overlap contigs**	**Full insert sequences**
Eye	Dj_aE	000	Plasmid	SK	ABI 3700	918*	-	-	-
Head	Dj_aH	000	Plasmid	SK, M13	ABI 3700	6,444*	3,163	689	495
Head	Dj_aH	001 - 022	Plasmid	SK	ABI 3700	5,024	-	-	21
Head	Dj_aH	101 - 140	Plasmid	SK	ABI 3700	2,364	-	-	4
Head	Dj_aH	201 - 227	TempliPhi	SK	ABI 3730xl	8,426	-	-	52
Head	Dj_aH	301 - 327	TempliPhi	SK, M13	ABI 3730xl	8,366	7,107	4,516	21
Head	Dj_aH	401 - 406	TempliPhi	T3	ABI 3730xl	2,056	-	-	-
Head	Dj_aH	501 - 530	TempliPhi	T3, M13	ABI 3730xl	9,462	8,191	5,888	-
Total						43,060	18,461	11,093	593

* DDBJ entries registered by previous research.

### *De novo* transcriptome assembly

**Figure 1:**
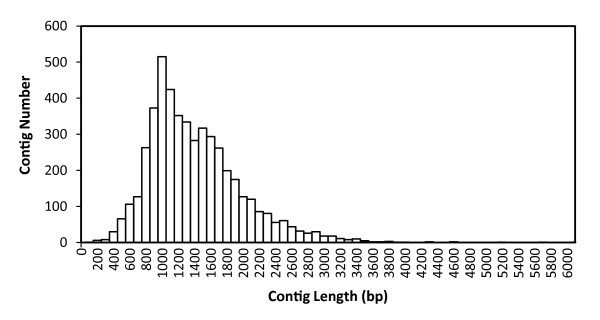
Distribution of number of sequences per contig in the unigenes.

**Figure 2:**
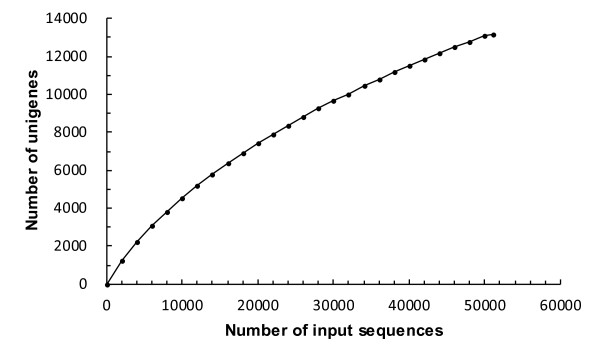
**Unigene accumulation curve.** The x-axis shows the number of EST sequences in the group used for unigene assembly, and the y-axis shows the number of unigenes consisting of contigs and singletons.

**Figure 3:**
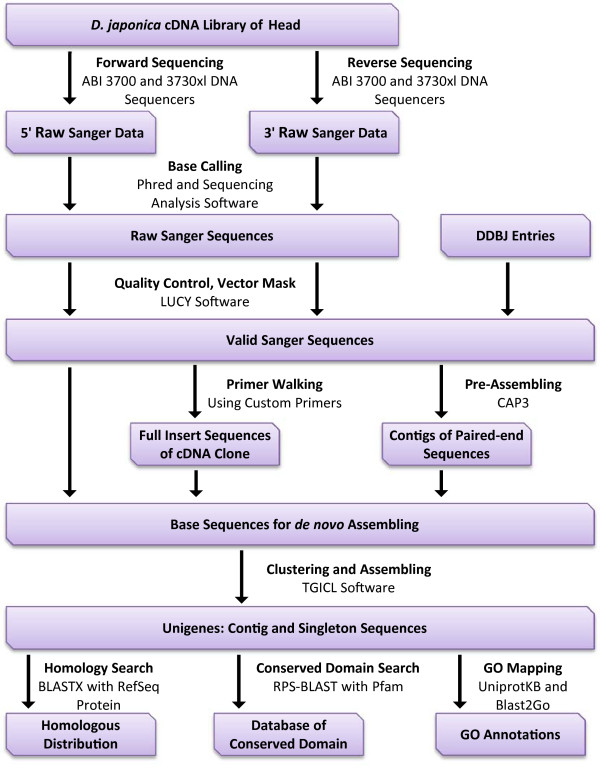
**Flow chart for the generation and annotation of *D. japonica *unigenes.**

Before *de novo* assembly, to generate accurate unigenes, 11,093 paired-assembly contigs were produced (Table 1) using paired-end sequences of the same clone and CAP3 assembler software [37]. After the addition of 7,362 DDBJ entries registered from previous research (Additional file 1), the complete sequence materials without the original reads that were members of paired-assembly contigs were further assembled into 4,883 contigs using TGICL software [38]. In addition, 8,284 sequences remained as singletons, resulting in a total of 13,167 unique sequences (Table 2). The average length of contigs was 1,360 bp, and the sum of all unique sequences was 12.6 Mbp, including singletons (Table 2). Figure 1 shows the distribution of the number of contigs with a particular length among the unigenes. The longest contig length was 6,040 bp. The histogram of contig depth showed that contigs with fewer than 4 copies and singletons accounted for 87% of unique sequences. In contrast, only 2 highly expressed contigs dominated the whole transcriptome sequences (Table 3). These profiles were consistent with the results of general non-normalized transcriptome analysis [39,40]. To estimate the transcriptome coverage for the data set, we assembled 2,000 replicate random sequences and calculated the non-redundant gene numbers (Figure 2). The workflow for the assembly construction process is shown in Figure 3.

**Table 2:** **Statistics of the *de novo *transcriptome assembly**

	**Number**	**Total base (bp)**	**Average length (bp)**	**Median (bp)**
Contigs	4,883	6,642,939	1360.4	1,252
Singletons	8,284	5,919,589	701.1	672
Unique sequences	13,167	12,562,528	940.5	846

Summary of the *de novo* transcriptome assembly using filter-passed and vector trimmed reads, pre-assembly contigs, and full-insert sequences.

**Table 3:** **Histogram of cluster sizes for *D. japonica *unigenes**

**Contig depth**	**Number of contigs**	**Percentage of unigenes**	**Integration**
1	8,488	63.81%	63.48%
2	1,686	12.67%	76.09%
3 - 4	1,423	10.70%	86.73%
5 - 8	922	6.93%	93.63%
9 - 16	471	3.54%	97.15%
17 - 32	217	1.63%	98.77%
33 - 64	96	0.72%	99.49%
65 - 128	46	0.35%	99.84%
129 - 256	17	0.13%	99.96%
257 - 512	3	0.02%	99.99%
513 - 1,024	0	0.00%	99.99%
1,025 - 2,048	1	0.01%	99.99%
2,049 - 4,096	1	0.01%	100.00%

The depth of contigs showing less than 5 copies of expression consist mostly of the unigene variation.

### Unigene annotations

**Figure 4:**
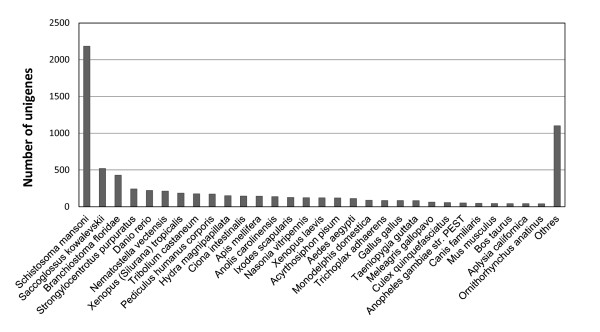
**Species distribution of significant homologous matches of the unique sequences.** A total of 7,334 sequences were hit with the RefSeq protein database using BLASTX with an E-value 1e-10. The top 30 species and number of sequences are shown.

The annotations of the *D. japonica* transcriptome were based on three types of approach: homology searching by BLAST [41], conserved protein domain detection [42], and Gene Ontology [35] classification. The BLASTX search against the NCBI Protein Reference Sequences database (RefSeq) [43,44] resulted in 7,334 (55.7%) unigene hits with significant similarity. The taxonomic distribution per organism using the best hit showed high similarity (2,184, 16.6%) with the schistosome, which belongs to the same phylum as planarians (Figure 4). Many planarian genes showed similarity to genes in not only the schistosome but also other organisms, including the hemichordate *S. kowalevskii* (520), chordate *B. floridae* (429), echinoderm *S. purpuratus* (242), and vertebrate *D. rerio* (221).

The conserved domain information for the transcriptome was obtained through the Pfam database [45] using RPS-BLAST, which scans a set of pre-calculated position-specific scoring matrices with a protein query. A total 4,609 (35.0%) conserved protein domains with 1,558 variations were confirmed in the complete set of unigenes. Protein kinase domains were the most frequent, with 307 hits, and the second- and third- most frequent domains were ankyrin repeats (101 hits) and RNA recognition motifs (71 hits) (Table 4). Domains with less than 5 hits consist mostly of the result (2,594 hits).

**Table 4:** **The top 40 Pfam domains and families in *D. japonica *unigenes**

**Pfam_ID**	**Conserved Domain**	**Genes**
pfam00069	Protein kinase domain	307
pfam12796	Ankyrin repeats	101
pfam00076	RNA recognition motif	71
pfam00071	Ras family	62
pfam07690	Major Facilitator Superfamily	49
pfam07714	Protein tyrosine kinase	38
pfam00876	Innexin	34
pfam00443	Ubiquitin carboxyl-terminal hydrolase	33
pfam00067	Cytochrome P450	32
pfam00270	DEAD/DEAH box helicase	29
pfam00102	Protein-tyrosine phosphatase	28
pfam00001	7 transmembrane receptor (rhodopsin family)	27
pfam00022	Actin	27
pfam00112	Papain family cysteine protease	27
pfam00350	Dynamin family	26
pfam00240	Ubiquitin family	23
pfam00335	Tetraspanin family	23
pfam11901	Protein of unknown function (DUF3421)	23
pfam03028	Dynein heavy chain and region D6 of dynein motor	22
pfam00226	DnaJ domain	21
pfam00271	Helicase conserved C-terminal domain	21
pfam00012	Hsp70 protein	20
pfam02931	Neurotransmitter-gated ion-channel ligand binding domain	20
pfam00153	Mitochondrial carrier protein	19
pfam00179	Ubiquitin-conjugating enzyme	19
pfam00620	RhoGAP domain	19
pfam01576	Myosin tail	19
pfam03953	Tubulin C-terminal domain	19
pfam00004	ATPase family associated with various cellular activities	16
pfam00017	SH2 domain	16
pfam00060	Ligand-gated ion channel	16
pfam00089	Trypsin	16
pfam00155	Aminotransferase class I and II	16
pfam00168	C2 domain	16
pfam01145	SPFH domain/Band 7 family	16
pfam00046	Homeobox domain	15
pfam00091	Tubulin/FtsZ family, GTPase domain	15
pfam00307	Calponin homology (CH) domain	15
pfam00501	AMP-binding enzyme	15
pfam00782	Dual specificity phosphatase, catalytic domain	15

To address the functional categories of the *D. japonica* transcriptome, all the unigenes were assigned a Gene Ontology (GO) classification based on BLASTX hits against the UniProtKB/Swiss-Prot database [46], which has reliable information for GO terms, and the annotation based on related studies. By referring to each GO term from the UniProt database, the terms associated with the unigenes were consolidated into higher classes using GO slim digestion [47] via software [48].

### Amino acid substitutions between two planarians

**Figure 5:**
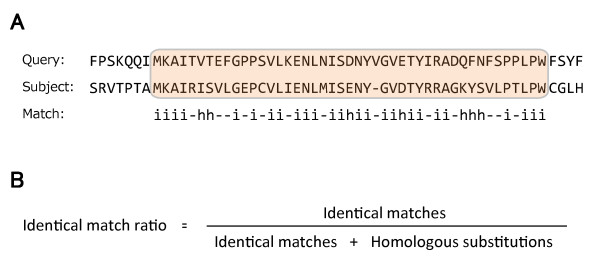
**Algorithm for the identical match ratio calculation between conserved proteins.** (**A**) Alignment output of Protein-BLAST. The colored box shows a conserved region between query and subject sequences defined by BLAST. Match type i and h indicate identical matches and homologous substitutions, which are based on the BLOSUM62 matrix, and - means no similarity or a gap. (**B**) The equation used to calculate the identical match ratio. A large substitution ratio within a conserved region leads to a decreased identical match ratio, which indicates that a homologous pair exhibits a high rate of diversification. In the case of A, the identical match ratio is "0.78".

The protein BLAST software identifies the conserved regions and the degrees of similarity between query and subject amino acid sequences. BLAST shows not only identical amino acids at a given position in the alignment, but also homologous substitutions, which are determined from the scoring matrix (Figure 5A). A method for calculating the identical match ratio (the number of identical matches divided by the sum of identical matches plus homologous substitutions) (Figure 5B) was applied to find strongly and weakly conserved proteins between the two planarians *D. japonica* and *S. mediterranea*. After comparative analysis of each unigene by TBLASTX, to obtain sufficient homologous pairs for the extraction, some filter options were applied to the results. Finally, a total of 3,177 pairs remained as homologous genes, and the top 15% (high substitution) and the bottom 15% (low substitution) of them regarding the identical match ratio (a total of 952 genes) were sorted into functional categories by RPS-BLAST analysis using the eukaryotic clusters of orthologous groups (KOG) database [49]. Of these, 843 (88.6%) genes were classified into 4 KOG categories with 24 KOG functions. No genes were classified as ‘Cell motility’ KOG function.

**Figure 6:**
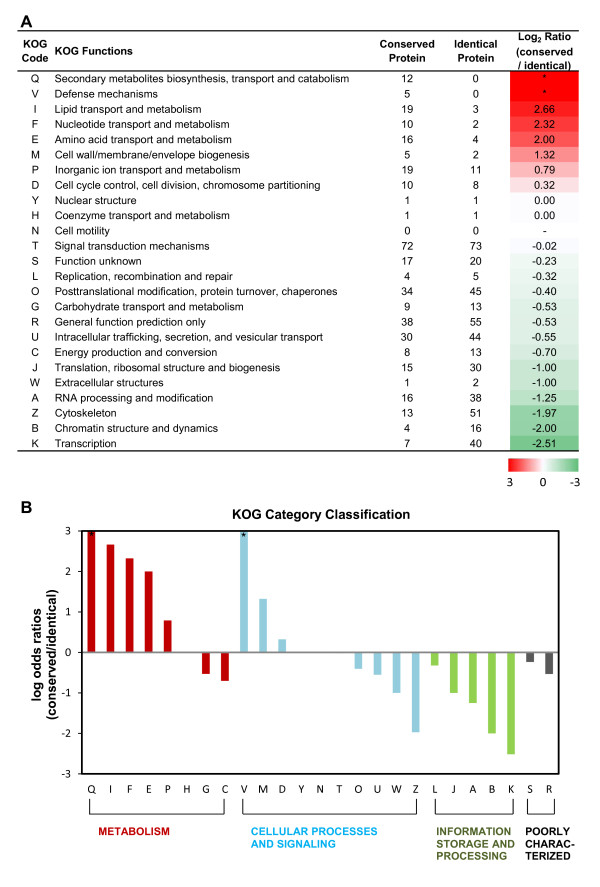
**Classification of the planarian conserved/identical proteins using KOG annotation.** (**A**) The definitions of conserved protein and identical protein were derived from the identical match ratio calculation using the BLOSUM62 substitution matrix and the conserved region between homologous proteins in the 2 planarian species which were predicted by protein BLAST. After conserved domain search using the KOG database, each gene was classified in accordance with KOG functions. The columns of Conserved and Identical proteins show the numbers of genes that were classified into each function. The heat plot shows the log_2_ conserved/identical match ratio, with red indicating a high proportion of proteins with substitutions and green indicating that the majority of proteins are identical for the indicated function. * indicates a function that contained only conserved proteins (shown in red color). (**B**) KOG category classification shows obvious patterns that were clearly distinguishable for each category. Many relatively conserved genes were concentrated in ‘Metabolism’, whereas ‘Information Storage and Processing’ contained many identical genes.

Figure 6A shows the classified gene number and the log_2_ odds ratios between conserved and identical gene pairs. The ratio varied widely (from +2.66 to −2.51) according to the KOG function, as did the number of genes, and showed a linear gradient with little bias. In the functions ‘Secondary metabolites biosynthesis, transport and catabolism’ and ‘Defense mechanisms’, there were only conserved group genes, as indicated by the identical match ratio. Inspection of the KOG category classification (Figure 6B) showed that many conserved genes were concentrated in the ‘Metabolism’ category, while ‘Information Storage and Processing’ contained many identical genes. In contrast, genes that were classified into ‘Defense mechanisms’ and ‘Cell wall/membrane/envelope biogenesis’ functions showed a low identical match ratio among genes in the ‘Cellular Processes and Signaling’ category. Additional file 2 displays examples of high-substitution-rate proteins between *D. japonica* and *S. mediterranea* for each category.

### Extraction of the planarian CNS-development genes

To obtain insight into the evolutionary origin of a functional brain, central nervous system (CNS)-development genes were extracted from the *D. japonica* unigenes using GO annotations as a common knowledge base. The category “CNS-development” was chosen here because we reasoned that the strong evolutionary/structural conservation of the planarian and higher animal brains implies conservation of many brain developmental pathways. Based on the results of BLASTX analysis against UniProtKB, each primary hit was defined as a putative planarian protein and function. The GO term ‘central nervous system development’ (GO:0007417) has 14 subcategories, each of which has a considerable number of subfamilies and terms. Because a protein which had multifunctions was mapped to each respective term separately, the total count of hits classed to CNS-development genes was increased by such duplications. The classification analysis produced a total of 96 matches and 82 CNS-development genes, with 6 subcategories (Table 5). The most highly represented subcategory was ‘brain development’ (GO:0007420), with 68 matches, and the second- and third-most frequent were ‘spinal cord development’ (GO:0021510) and ‘central nervous system neuron differentiation’ (GO:0021953), with 10 and 8 matches, respectively. In addition, 4 matches were found for the subcategory ‘ventral cord development’.

**Table 5:** **Comparative analysis of *D. japonica *CNS development genes with *S. mediterranea *and the schistosoma**

**GO Term**	***D. japonica*Unigenes**	**UniProt KB Homology**	**UniProt E-value**	***S. mediterranea***		**Schistosoma**	
				**Genome**	**mRNA**	**Genome**	**mRNA**
**ventral cord development**	Dj_CL2868_001_b2	Protein numb	2.00E-68	**+**	**+**	**+**	**+**
	Dj_CL0775_001_b2	Ras-related protein Rac1	2.00E-85	**+**	**+**	**+**	**+**
	Dj_aH_314_P04.full	Protein nubbin	7.00E-21	**+**	**+**	**+**	
	Dj_aH_000_00626HN.full	Zinc finger protein jing	1.00E-12	**+**	**+**	**+**	
**brain development**	Dj_CL0266_002_b2	Dynein heavy chain, cytoplasmic	0.00E + 00	**+**	**+**	**+**	**+**
	Dj_aH_323_P16	DNA topoisomerase 2-beta	1.00E-129	**+**	**+**	**+**	**+**
	Dj_CL2992_001_b2	Probable global transcription activator SNF2L1	1.00E-104	**+**	**+**	**+**	**+**
	Dj_CL0800_001_b2	Transcriptional regulator ATRX	5.00E-84	**+**	**+**	**+**	**+**
	Dj_aH_133136_J24	Myosin-10	2.00E-80	**+**	**+**	**+**	**+**
	Dj_CL1927_001_b2	Protein Wnt-4	3.00E-62	**+**	**+**	**+**	**+**
	Dj_CL1142_001_b2	Brain tumor protein	5.00E-35	**+**	**+**	**+**	**+**
	Dj_CL2438_001_b2	Ubiquitin carboxyl-terminal hydrolase isozyme L5	3.00E-72	**+**	**+**	**+**	**+**
	Dj_CL1575_001_b2	Alpha-soluble NSF attachment protein	1.00E-77	**+**	**+**	**+**	**+**
	Dj_aH_121124_O06	cGMP-dependent protein kinase 1	4.00E-81	**+**	**+**	**+**	**+**
	Dj_aH_203_F09	Neurofibromin	2.00E-40	**+**	**+**	**+**	**+**
	Dj_aH_517_H15.double	Receptor tyrosine-protein kinase erbB-4	3.00E-83	**+**	**+**	**+**	**+**
	Dj_aH_526_D23.double	Hypoxanthine-guanine phosphoribosyltransferase	4.00E-61	**+**	**+**	**+**	**+**
	Dj_aH_214_D06.full	Homeobox protein SIX3	2.00E-90	**+**	**+**	**+**	**+**
	Dj_aH_202_C21	Plasma membrane calcium-transporting ATPase 3	8.00E-47	**+**	**+**	**+**	**+**
	Dj_CL0788_001_b2	Neural cell adhesion molecule 1	5.00E-18	**+**	**+**	**+**	**+**
	Dj_aH_204_E12	Protein kinase C iota type	1.00E-49	**+**	**+**	**+**	**+**
	Dj_CL3335_001_b2	Nuclear factor 1 B-type	4.00E-14	**+**	**+**	**+**	**+**
	Dj_aH_307_L04.rev	cGMP-dependent protein kinase 1	8.00E-55	**+**	**+**	**+**	**+**
	Dj_CL3457_001_b2	RNA binding protein fox-1 homolog 1	5.00E-33	**+**	**+**	**+**	**+**
	Dj_CL1765_001_b2	Zinc finger protein ZIC 2	2.00E-73	**+**	**+**	**+**	**+**
	Dj_aH_503_H01	Epidermal growth factor receptor	9.00E-38	**+**	**+**	**+**	**+**
	Dj_aH_511_L17	Tubby-related protein 3	2.00E-44	**+**	**+**	**+**	**+**
	Dj_CL1678_001_b2	NADH dehydrogenase [ubiquinone] iron-sulfur protein 4, mitochondrial	2.00E-35	**+**	**+**	**+**	**+**
	Dj_CL2162_001_b2	Menin	1.00E-37	**+**	**+**	**+**	**+**
	Dj_CL3785_001_b2	Excitatory amino acid transporter 2	5.00E-28	**+**	**+**	**+**	**+**
	Dj_aH_514_N16	DNA topoisomerase 2-beta	2.00E-33	**+**	**+**	**+**	**+**
	Dj_aH_325_L12.double	Paired mesoderm homeobox protein 2B	2.00E-28	**+**	**+**	**+**	**+**
	Dj_aH_305_P01	Transcriptional regulator ATRX	9.00E-29	**+**	**+**	**+**	**+**
	Dj_aH_520_E14	Epidermal growth factor receptor	9.00E-15	**+**	**+**	**+**	**+**
	Dj_aH_000_01325HH.double	NADH dehydrogenase [ubiquinone] iron-sulfur protein 4, mitochondrial	1.00E-24	**+**	**+**	**+**	**+**
	Dj_aH_217_D18	Cysteine string protein	7.00E-28	**+**	**+**	**+**	**+**
	Dj_aH_402_H01	LIM homeobox transcription factor 1-alpha	2.00E-29	**+**	**+**	**+**	**+**
	Dj_CL3423_001_b2	Lethal(2) giant larvae protein homolog 1	2.00E-14	**+**	**+**	**+**	**+**
	Dj_aH_222_K23	Leishmanolysin-like peptidase	2.00E-38	**+**	**+**	**+**	**+**
	Dj_aH_505_J10	Ski oncogene	1.00E-32	**+**	**+**	**+**	**+**
	Dj_aH_519_D15.double	Bardet-Biedl syndrome 2 protein homolog	4.00E-51	**+**	**+**	**+**	**+**
	Dj_CL0001_086_b2	Paired box protein Pax-6	5.00E-72	**+**	**+**	**+**	**+**
	Dj_CL3673_001_b2	Glutamate [NMDA] receptor subunit epsilon-1	2.00E-37	**+**	**+**	**+**	**+**
	Dj_aH_000_04532HH.full	Protocadherin-18	3.00E-39	**+**		**+**	**+**
	Dj_aH_523_G03.rev	Tubby-related protein 3	4.00E-23	**+**	**+**	**+**	**+**
	Dj_CL1956_001_b2	Homeobox protein meis3-A	3.00E-44	**+**	**+**	**+**	**+**
	Dj_CL1514_001_b2	Protocadherin-like wing polarity protein stan	2.00E-20	**+**	**+**	**+**	**+**
	Dj_CL2141_001_b2	Large proline-rich protein BAG6	2.00E-11	**+**	**+**	**+**	**+**
	Dj_aH_306_A21	Sphingosine kinase 2	3.00E-14	**+**	**+**	**+**	
	Dj_CL3244_001_b2	Neuroligin-4, X-linked	1.00E-21	**+**	**+**	**+**	
	Dj_CL4115_001_b2	Neuroglian	2.00E-41	**+**	**+**	**+**	
	Dj_aH_306_H01	Protein phosphatase Slingshot	6.00E-14	**+**	**+**	**+**	
	Dj_CL1452_001_b2	SH3 and multiple ankyrin repeat domains protein 2	3.00E-18	**+**	**+**	**+**	
	Dj_CL1757_001_b2	Intraflagellar transport protein 88 homolog	4.00E-74	**+**	**+**	**+**	
	Dj_aH_527_L21	Cytosolic carboxypeptidase 1	3.00E-76	**+**	**+**	**+**	
	Dj_aH_522_B06	Slit homolog 2 protein (Fragment)	7.00E-15	**+**	**+**	**+**	
	Dj_aH_137140_C22	Bardet-Biedl syndrome 4 protein	6.00E-47	**+**	**+**	**+**	
	Dj_aH_303_K17	Zinc finger protein Dzip1	1.00E-33	**+**	**+**	**+**	
	Dj_aH_207_O05	Potassium/sodium hyperpolarization-activated cyclic nucleotide-gated channel 2	8.00E-88	**+**	**+**	**+**	
	Dj_CL0854_001_b2	Secreted frizzled-related protein 5	3.00E-29	**+**	**+**	**+**	
	Dj_CL3133_001_b2	Cytosolic carboxypeptidase 1	3.00E-35	**+**	**+**	**+**	
	Dj_aH_313_M09.double	Cyclin-dependent kinase 5 activator 1	2.00E-48	**+**	**+**	**+**	
	Dj_CL0876_001_b2	Sodium/calcium exchanger 1	1.00E-161	**+**	**+**	**+**	
	Dj_aH_303_F09	Frizzled-8	1.00E-21	**+**	**+**	**+**	
	Dj_CL4143_001_b2	Intraflagellar transport protein 88 homolog	2.00E-81	**+**	**+**	**+**	
	Dj_aH_530_O19	Paired box protein Pax-6	9.00E-55	**+**	**+**	**+**	
	Dj_aH_007_F21	Protein Hook homolog 3	6.00E-13	**+**	**+**		
	Dj_aH_309_N15.double	Dixin	6.00E-11	**+**	**+**		
	Dj_CL4393_001_b2	Endothelin-converting enzyme 2	7.00E-34	**+**	**+**		
	Dj_aH_325_L06	Centrosomal protein of 290 kDa	3.00E-31	**+**	**+**		
	Dj_CL0234_001_b2	Reticulon-4	2.00E-13	**+**	**+**		
	Dj_aH_518_F04.double	Ceroid-lipofuscinosis neuronal protein 5	2.00E-74	**+**	**+**		
**spinal cord development**	Dj_aH_203_F09	Neurofibromin	2.00E-40	**+**	**+**	**+**	**+**
	Dj_aH_000_02581HH	Calpain-A	1.00E-51	**+**	**+**	**+**	**+**
	Dj_aH_502_D11	Calpain-A	2.00E-67	**+**	**+**	**+**	**+**
	Dj_aH_511_L17	Tubby-related protein 3	2.00E-44	**+**	**+**	**+**	**+**
	Dj_CL3713_001_b2	Suppressor of fused homolog	2.00E-21	**+**	**+**	**+**	**+**
	Dj_aH_000_01210HH.full	Suppressor of fused homolog	3.00E-15	**+**	**+**	**+**	**+**
	Dj_CL3673_001_b2	Glutamate [NMDA] receptor subunit epsilon-1	2.00E-37	**+**	**+**	**+**	**+**
	Dj_aH_523_G03.rev	Tubby-related protein 3	4.00E-23	**+**	**+**	**+**	**+**
	Dj_aH_522_B06	Slit homolog 2 protein (Fragment)	7.00E-15	**+**	**+**	**+**	
	Dj_aH_000_03614HH	DNA-binding protein SMUBP-2	4.00E-23	**+**	**+**		
**central nervous system neuron differentiation**	Dj_CL2485_001_b2	Spastin	1.00E-83	**+**	**+**	**+**	**+**
	Dj_aH_526_D23.double	Hypoxanthine-guanine phosphoribosyltransferase	4.00E-61	**+**	**+**	**+**	**+**
	Dj_aH_511_L17	Tubby-related protein 3	2.00E-44	**+**	**+**	**+**	**+**
	Dj_aH_402_H01	LIM homeobox transcription factor 1-alpha	2.00E-29	**+**	**+**	**+**	**+**
	Dj_aH_523_G03.rev	Tubby-related protein 3	4.00E-23	**+**	**+**	**+**	**+**
	Dj_aH_527_L21	Cytosolic carboxypeptidase 1	3.00E-76	**+**	**+**	**+**	
	Dj_CL3133_001_b2	Cytosolic carboxypeptidase 1	3.00E-35	**+**	**+**	**+**	
	Dj_aH_000_03614HH	DNA-binding protein SMUBP-2	4.00E-23	**+**	**+**		
**astrocyte differentiation**	Dj_aH_203_F09	Neurofibromin	2.00E-40	**+**	**+**	**+**	**+**
	Dj_CL0341_002_b2	Porphobilinogen deaminase	4.00E-69	**+**	**+**	**+**	**+**
	Dj_CL0341_001_b2	Porphobilinogen deaminase	2.00E-64	**+**	**+**	**+**	**+**
**oligodendrocyte differentiation**	Dj_aH_203_F09	Neurofibromin	2.00E-40	**+**	**+**	**+**	**+**
	Dj_aH_513_H15.rev	Exocyst complex component 4	7.00E-18	**+**	**+**	**+**	**+**
	Dj_CL1495_001_b2	Translation initiation factor eIF-2B subunit delta	4.00E-42	**+**	**+**	**+**	**+**

+ indicate the presence of gene which homologous with *D. japonica* CNS-development gene in *S. mediterranea* and the schistosoma.

### Comparative analysis of CNS-development genes among platyhelminths

Two types of schistosome genome dataset, the primary genome sequence and predicted protein sequences based on the genome sequence, and the primary genome sequence of *S. mediterranea,* were used for exploring the Platyhelminthes genes that are homologous with *D. japonica* CNS-development genes. The primary genome sequence provides evidence useful for distinguishing between genes with low expression and deleted genes. The predicted protein sequences were directly dependent on the computational process, and were not tested regarding whether they were supported by biological evidence of the gene expression, but contributed to the detection of genes that have small exons split by long intron regions. In the case of direct searching against primary genome sequences, the *D. japonica* unigenes were mapped by BLAT software [50] using the parameters of the six translation frames to compare the protein-protein sequences. In addition, the predicted protein sequences were analyzed using the TBLASTN program. The use of translated nucleotide databases and translated nucleotide query enables absorption of the third codon position mutations, and therefore it is useful for comparison of evolutionarily distant genes. In these searches, if either one of the two scores exceeded the threshold, the matched gene was defined as conserved. A transcriptome-transcriptome analysis among platyhelminths was performed using TBLASTX rather than BLASTN for the same reason as described above.

The summary showed that the *D. japonica* CNS-development genes were highly conserved among platyhelminths (Table 5). In the case of comparison with *S. mediterranea*, all of the 82 genes were detected in the genome sequence, and almost all of the genes (81/82) were found in the transcriptome data set obtained by next generation sequencing. In total, 75/82 (91%) *D. japonica* CNS-development genes were conserved in the schistosome genome. Most of these genes were in subcategories other than brain development, suggesting that the CNS-development genes were well conserved during evolution. In contrast to the high genome concordance, the results of transcriptome-transcriptome analysis showed a low concordance rate of 54/82 (66%), excluding genes in the categories ‘astrocyte differentiation’ and ‘oligodendrocyte differentiation’.

## Discussion

### *D. japonica* transcriptome

We performed large scale *D. japonica* transcriptome analysis using non-normalized cDNA libraries constructed from head tissue to construct a transcriptome database for comparative genomics and studies of brain evolution. Compared with normalized cDNA libraries, in which the proportion of high-copy housekeeping genes is decreased, non-normalized cDNA libraries have many redundant transcripts, but they keep the information of the original expression levels and are expected to provide raw expression profiles [40]. Next generation sequencing is a powerful tool that has provided massive datasets for genome and transcriptome analysis, including some datasets for the *S. mediterranea* transcriptome [26-28]. However, for comparative analysis between two planarians, it is important to use long and gapless consensus sequences of conserved proteins. To obtain such sequences, next-generation sequencing is not always the best tool. For example, Roche 454 cannot correctly read homo-polymer sequences like AAAA or TTTT (and this is relevant because planarians are AT-rich organisms), and this limitation may cause many frame-shift errors in a gene sequence. Also, although Illumina HiSeq generates a large volume of reads, they are rather short, and this makes it difficult to obtain long and correct contig sequences without high quality reference information. The paired-end application is a useful way to link separated contigs, but long gaps often arise when there are no consensus sequences. In contrast, Sanger sequencing provides long, high-quality mRNA reads, which are important for comparative analysis using consensus sequences of conserved protein domains. Furthermore, long reads can help to distinguish between paralogues and alternative splicing variants, and thus are useful for transcriptome research of non-model organisms for which high-quality genomic information is lacking.

In this study, we obtained a total of 13,167 unigenes with 940.5 bp average length as a result of *de novo* assembly using 54,752 newly acquired long Sanger sequences. Two types of pre-assembled reads, gap-closed sequences obtained by a primer walking method, and overlap-joined contigs from the same clone, contributed to extending the contig length. Additionally, many singletons that were unassembled ESTs also had sufficient length to identify the function. The histogram of assembly statistics (Table 3) and functional annotations of the assembled unigenes, based on conserved domain search by RPS-BLAST (Table 4), indicated that we had built an encompassing and low-bias gene profile that provided enough information for comparative transcriptome analysis among two planarians and a schistosome. Regarding approaches to non-model organism transcriptomes, the algorithms applied and dataset selection are of the same importance as *de novo* assembling for finding new evolutionary events or pathways. Here, we report two different approaches to finding such events, not simply the acquisition of basic knowledge about the *D. japonica* transcriptome.

### Comparative analysis between two planarians

The acquisitions of gene mutations and corresponding amino acid substitutions are not random events, but rather are affected by the pressure of natural selection. It is generally accepted that similar proteins with homologous substitutions share basic molecular functions in organisms [51]. The biotic features (lifestyle, ability to regenerate, and cell characteristics) of the two planarians studied here, *D. japonica* and *S. mediterranea*, are quite similar, and large numbers of their genes and their encoded amino acid sequences are well conserved. However, the nucleic acid sequences of many homologous genes have low similarity between these two planarians, and there are many genes containing homologous substitutions for amino acids in conserved regions, although the profiles and details remain unknown. For example, PIWI2 alignment shows 94% identical matches of amino acids over the entire sequence ( Additional file 3). But in the case of GGT1, only 70% of amino acids are the same, although the percentage rises to 88% if homologous substitutions are included ( Additional file 4).

Based on the hypothesis that proteins with many substitutable amino acids could be candidates for members of gene groups that are able to undergo changes to accommodate to different environments, we developed a new method for detecting the fraction of substitutable amino acids within individual genes. To view the trends in functional groups of proteins rather than in each gene, we classified the results calculated by this method into functions and categories based on KOG annotation. The log_2_ ratio between the number of proteins with homologous substitutions and the number with identical matches revealed distinctive features of function and category (Figure 6). Although a very long time has passed since their divergence from their common ancestor, amino acids in proteins involved in basal mechanisms such as functions of the ‘Information Storage and Processing’ category and cellular structure-related functions ‘Cytoskeleton’ and ‘Extracellular structures’ have not undergone replacement even by amino acids that have similar biochemical properties, presumably as a result of selection pressure. By contrast, in the function ‘Defense mechanisms’, which is directly linked to an organism’s environment, every gene mapped to this function displayed a high rate of substitution. Among the proteins in the category ‘Metabolism’, the energy metabolism functions ‘Carbohydrate transport and metabolism’ and ‘Energy production and conversion’ showed low rates of substitution, whereas genes related to digestion processes (lipid, nucleotide and amino acid digestion) had high rates of substitution, perhaps reflecting responsiveness to their feeding habitat. It is interesting that many accumulations of amino-acid substitutions were observed in proteins that were well conserved not only between the two planarians examined here, but among many species, and were expected to maintain their biological function (Additional file 2). Previous studies of schistosomes suggested that these animals are incapable of *de novo* synthesis of sterols or free fatty acids and must use precursors of fatty acids from the host [19,52]. It could be considered that the effects of selection pressure on metabolism-related genes are decreased in parasitic life, because an appropriate nutritional environment is provided by the host. These previously reported results support our hypothesis that, in addition to selective pressure from the environment, the potential of genes to accommodate substitutions is also an important factor in evolution.

### Evolutionary conservation of CNS-development genes

As mentioned above, platyhelminths are considered to be primitive animals that possess a functional CNS closely related to the evolutionary origin of the CNS. The *D. japonica* CNS is composed of an anterior cephalic ganglion and ventral nerve cords, which are morphologically distinguishable structures. Planarians can sense light and chemical signals transmitted through their eyes and chemosensors, respectively, to their brain, and show different behaviors according to the type of signal.

In addition the partial results of previous transcriptome analysis, we previously reported several genes specifically expressed in the CNS, such as synaptotagmin, prohormone convertase 2, and netrin [7,9,34]. But, as compared with model organisms, the gene information for platyhelminths is very limited, and this makes it difficult to perform whole transcriptome comparison. For this reason we used Gene Ontology and the category “CNS-development”, which is based on information across many species, including vertebrates and non-vertebrates. We think this annotation can serve well as a common platform for comparing and annotating among platyhelminths. Both the functionality and complexity of the planarian CNS are supported by our identification of 82 *D. japonica* genes related to CNS development, based on the Gene Ontology and UniProtKB knowledge base. Homology search with *S. mediterranea* showed that the genes are quite highly conserved between these two planarian species. All of the 82 genes were found in the *S. mediterranea* genome sequence and almost all of these genes (81/82) were found in the *S. mediterranea* transcriptome data set derived by next generation sequencing. This number of CNS-development-related genes is not much lower than that in model organisms known to have highly functional brains, namely, *D. melanogaster* (228), *D. rerio* (285), *G. gallus* (129) and *M. musculus* (487) [35]. In addition, these numbers are clearly larger than the number of CNS-development-related genes in *C. elegans* (6), which may have lost a complex CNS, and suggest the possibility that the planarian has a functional brain at not only the developmental level but also the genomic level.

The CNS of *S. mansoni* consists of a central ganglion, six pairs of main nerve trunks, and longitudinal nerve cords, which extend along the length of the body [4]. Additionally, six different types of sensory papillae are known, and are considered to be valuable structures for penetration and navigation through the vasculature in the body of the host. Using the predicted protein sequences of the schistosome and performing an exhaustive search of all possible CNS-development genes, 75 homologs of the 82 genes in *D. japonica* were successfully detected. This result indicates that the planarian CNS-development genes already existed before the divergence of planarians and schistosomes from their last common ancestor. Interestingly, expression of about one-third of the CNS genes was not observed in the schistosome Unigene database, which was built using transcript sequences. Because the schistosome unigenes were constructed from 7 different developmental stages, 1,611 libraries, and 152,704 sequences, their completeness is considered to be extremely high, and therefore these CNS-development genes not observed in this schistosome database can be concluded to have extremely low or no expression. It is possible that, in contrast to the free-living lifestyle of planarians, the parasitic life of schistosomes resulted in selective degeneration of unnecessary genes in schistosomes. The features of the brain are determined to a large degree by the differential regulation of the transcriptome during an animal’s life cycle. Schistosomes not only inhabit various tissues of their intermediate and definitive hosts, but also traverse freshwater environments to change hosts. Viewed in this light, it might have been expected that the schistosome would have had more gene components related to the CNS, but instead it showed a reduced number of such genes. This might be because planarians face more complex circumstances than schistosomes regarding enemies or climate throughout their lifetime, or because schistosomes construct complicated components using more limited gene sets. Further studies will be needed to clarify the relation between gene diversification and external factors in organisms’ environments. To validate possible scenarios of planarian CNS evolution, this database will be valuable for the identification of relevant genes. More extensive analyses of genes and genomic resources will be needed to better understand the functions and evolution of the CNS.

## Conclusions

We have produced 54,752 high-quality EST reads from a head library of the planarian *D. japonica*, and *de novo* assembly analysis of these ESTs produced 13,167 unigene sequences. Similarity search against public databases and conserved domain analysis of predicted protein sequences indicated that this dataset is a useful resource for comparative transcriptome studies. Comparison of homologous genes between two planarian species led us to hypothesize that not only the pressure of natural selection but also the potential of genes is important for an acceleration of the accumulation of amino-acid substitutions, a hypothesis that is supported by previous studies showing that schistosomes are defective in lipid metabolism.

A total of 82 planarian CNS-development genes were extracted using Gene Ontology annotation, and this result suggested the possibility that the planarian has a functional brain both developmentally and genetically. Mapping of these genes onto the schistosome genome showed that the 91% of the planarian CNS-development genes were conserved within the schistosome genome. However, approximately one-third of the planarian CNS genes were not expressed in the schistosome. These analyses suggest that the establishment of the planarian CNS occurred before the divergence of planarians from their common ancestor with schistosomes, but that these two genuses subsequently diversified to adapt to their differing circumstances regarding the complexity needed for a free versus a parasitic life. This database of the *D. japonica* transcriptome constructed here provides an important resource not only for planarian research, but also for comparative analyses of the CNS.

## Methods

### Animal materials

An asexual clonal strain of the planarian *Dugesia japonica* derived from the Iruma River in Gifu prefecture, Japan, was used. This strain is named the GI strain. Intact animals were maintained in autoclaved tap water at 22–24 °C. More than 500 planarians of length 5–7 mm that had starved for 7–10 days were used in this study. After amputation at the prepharyngeal region under a phase-contrast microscope, the head fragments were collected to construct the head cDNA library.

### cDNA library construction and DNA sequencing

PolyA + RNAs were isolated from the head fragments and cloned into Uni-ZAP XR vector (Stratagene) according to the manufacturer’s instructions. Using a Gigapack III Gold Cloning kit (Stratagene), the vector-containing cDNAs were packaged into lambda phage. The clones were converted into pBluescript SK(−) phagemids, and transformed into XL1-Blue MRF strain (Stratagene). The bacterial colonies were randomly picked using a colony picker QPix (GENETIX), and were grown overnight with ampicillin. The template DNAs for the 000–140 series (Table 1) were prepared using MultiScreen-NA and FB plates (Millipore). In the case of the 201–530 series, the library clone DNAs were amplified using the TempliPhi reaction, based on ϕ29 rolling circle replication of DNA (GE Healthcare). The sequencing reaction was performed using a BigDye terminator v.3.1 cycle sequencing kit (Life Technologies). The primers used in the reaction were SK and T3 for the forward direction, and M13 for the reverse direction. The sequencing reaction products were analyzed using a PRISM 3700 and 3730xl DNA analyzer (Life Technologies). The convention for naming of EST sequences is: [Library prefix]_[Plate number]_[ID]. The sequence name extensions, no-extension, ‘.rev’, ‘.double’ and ‘.full’, mean forward-read, reverse-read, paired-assembly contig and gap-closed sequence, respectively. Dj_CL means contig sequence.

### Sequence validation

The base calling for 000–140 series sequences was processed using Phred software, and other series were base called using Sequencing Analysis Software ver.5.2 (Life Technologies) with KB Basecaller (Life Technologies). After base calling, lower quality regions and vector sequences were trimmed using LUCY software (ver.1.19p) [53] with quality threshold of 0.01. Full-insert cDNA sequences were obtained by a primer-walking sequencing method until the sequence of both edges of the insert had been determined.

### *De novo* assembly

Before whole *de novo* assembly, we used CAP3 software (ver. 04/15/05) to assemble the 5’- and 3’-end sequences of the same clone in the ESTs. In addition, 918 eye and 6,444 head EST entries were obtained from DDBJ (Additional file 1). To construct unigene sequences, all resources for EST sequences were clustered and assembled based on sequence similarity to generate a consensus sequence using TGICL software (ver. 2.1) with ‘-n 10000 -p 85 -l 60 -v 40’ parameters.

### Homology and conserved domain search of *D. japonica* unigenes

A survey of taxonomic distribution was conducted by matching the EST unigenes to the RefSeq protein database (Jun 5, 2011) using BLASTX software (ver. 2.2.23) with 1e-10 threshold. Only the top hit and the information on species were extracted and totaled from those results. Protein domain searches were performed with RPS-BLAST software (ver. 2.2.23) against the Pfam database (ver. 25.0) using the best hit with an E-value < 1e-10.

### Classification of identical/conserved proteins using KOG annotation

The evolutionarily shared gene pairs and the conserved regions between two planarians, *D. japonica* and *S. mediterranea*, were searched using the TBLASTX program against *S. mediterranea* unigenes (Build #4) with the following filter options: BLOSUM62 substitution matrix, sequence length of *D. japonica* unigene ≧600 bp, 1e-30 threshold and size of conserved region ≧80 bp. Each conserved region reported by TBLASTX was analyzed to measure the identical match ratio (Figure 5) to determine whether the protein was a high- or low-substitution protein. The KOG database and RPS-BLAST software were used to classify the genes with E-value less than 1e-10 into KOG functions and categories.

### Gene ontology classification

To obtain reliable annotation for GO classification, we chose the UniProtKB/Swiss-Prot database (release 2011_06), which is a high-quality manually annotated and non-redundant protein sequence dataset. After BLASTX analysis with 1e-10 threshold, the top BLAST hit was used as a putative protein name of the input unigene sequence. Ontologies of the UniProt knowledge base were used for the conversion of the protein to GO terms, and GO slim digestion was performed to get a broad overview of the ontology content without the details of the specific segmentalized terms.

### Exhaustive comparative analysis of *D. japonica* CNS-development genes with *S. mediterranea* and schistosome genes

Using the previous GO annotations of the unigenes, the genes that had GO term ‘central nervous system development’ (GO:0007417) or its 14 subcategories were defined as CNS-development genes. The genes that were defined by descendant terms of the subcategories of CNS development were added to the ancestral category to clarify the composition of planarian CNS genes. Comparison of *D. japonica* unigenes and the schistosome genome was performed using 2 pairs of software and the schistosome database with 1e-10 threshold: TBLASX (ver. 2.2.23) with the predicted protein (ver. 4) database [54], and BLAT software (ver. 3.4) with super contigs of genome sequences (ver. 3.1), respectively. If either one passed the threshold, the gene was defined as evolutionarily conserved. In cases of comparison of gene expression between *D. japonica* and the schistosome, TBLASTN (ver. 2.2.23) software and the schistosome unigenes (Build #19) were used and the same threshold was set for comparison. The same methods as those used for the schistosome were applied to comparative analysis of *D. japonica* with *S. mediterranea* using threshold1e-30, the super contigs of genome sequences (ver. 3.1) [55] and transcriptome resources [26,27].

## Abbreviations

cDNA: Complementary DNA; CNS: Central nervous system; EST: Expressed sequence tag; FACS: Fluorescence activated cell sorting; GO: Gene ontology; KOG: Eukaryotic clusters of orthologous groups; qRT-PCR: Quantitative reverse transcription polymerase chain reaction; RefSeq: NCBI reference sequence.

## Competing interests

The authors declare that they have no competing interests.

## Authors’ contributions

ON performed bioinformatics analysis and wrote the manuscript. YH performed cDNA library cloning and DNA sequencing. HT designed the system for large scale DNA sequencing. KA coordinated the EST project and designed the study. All authors read and approved the final manuscript.

## Supplementary Material

Additional file 1 7,362 DDBJ entries registered by previous research. Click here for file

Additional file 2 **Examples of high-substitution proteins between *D. japonica *and *S. mediterranea.*** Click here for file

Additional file 3 **Alignment of *D. japonica *and *S. mediterranea *PIWI2.** Click here for file

Additional file 4 **Alignment of *D. japonica *and *S. mediterranea *GGT1.** Click here for file
