# Supplementary material for: Comparative transcriptome analysis between planarian Dugesia japonica and other platyhelminth species
Source: BMC Genomics. 2012 Jun 29;13:289. doi: 10.1186/1471-2164-13-289 (PMC3507646; doi:10.1186/1471-2164-13-289)
Supplement: Additional file 2 — Examples of high-substitution proteins between D. japonica and S. mediterranea. [file 1471-2164-13-289-S2.pdf]

Additional file 4. Examples of high-substitution proteins between *D. japonica* and *S. mediterranea*

KOG Functions	*D. japonica* Unigenes	KOG Description
Secondary metabolites biosynthesis, transport and catabolism	Dj_CL1786_001_b2	Glutathione synthetase
	Dj_aH_000_04635HH.full	Amine oxidase
	Dj_aH_323_I13.rev	Transporter, ABC superfamily
	Dj_CL0815_001_b2	Pristanoyl-CoA/acyl-CoA oxidase
	Dj_CL0185_004_b2	Cytochrome P450 CYP2 subfamily
	Dj_aH_516_K24.double	Cytochrome P450 CYP3/CYP5/CYP6/CYP9 subfamilies
	Dj_aH_528_M20.rev	Hydroxysteroid 17-beta dehydrogenase 11
Defense mechanisms	Dj_aH_306_H01	Dual specificity phosphatase
	Dj_CL2549_001_b2	B-cell receptor-associated protein and related proteins
	Dj_CL1026_001_b2	Serpin
	Dj_CL3487_001_b2	Complement component 1, Q subcomponent binding protein/mRNA splicing factor SF2, subunit P32
Lipid transport and metabolism	Dj_CL0815_001_b2	Pristanoyl-CoA/acyl-CoA oxidase
	Dj_CL1029_001_b2	Very-long-chain acyl-CoA dehydrogenase
	Dj_CL4018_001_b2	Lysophosphatidic acid acyltransferase endophilin/SH3GL, involved in synaptic vesicle formation
	Dj_aH_000_04081HH.rev	Acyl-CoA synthetase
	Dj_aH_501_N03.rev	Amidases
	Dj_CL0322_001_b2	Long-chain acyl-CoA synthetases (AMP-forming)
	Dj_aH_109112_E24	Hydroxymethylglutaryl-CoA synthase
	Dj_CL1430_001_b2	Phosphatidylinositol transfer protein SEC14 and related proteins
	Dj_CL0981_001_b2	Mevalonate kinase MVK/ERG12
	Dj_CL4218_001_b2	Enoyl-CoA isomerase
	Dj_CL1268_001_b2	Triglyceride lipase-cholesterol esterase
	Dj_aH_226_J12	Predicted undecaprenyl diphosphate synthase
	Dj_CL3958_001_b2	Lipid phosphate phosphatase and related enzymes of the PAP2 family
	Dj_CL3075_001_b2	Phospholipase
	Dj_CL0940_001_b2	Carnitine O-acyltransferase CRAT
	Dj_CL2702_001_b2	Soluble epoxide hydrolase
Nucleotide transport and metabolism	Dj_CL1377_001_b2	Glutamine phosphoribosylpyrophosphate amidotransferase
	Dj_aH_325_K24.rev	Ribonucleotide reductase, alpha subunit
	Dj_aH_304_D04.double	Nucleoside phosphatase
	Dj_CL2998_001_b2	Nucleoside transporter
	Dj_aH_000_00767HH.rev	Phosphoribosylformylglycinamidine synthase
	Dj_aH_222_B06	Adenylate kinase
	Dj_aH_522_G08.double	Uridylate kinase/adenylate kinase
	Dj_aH_530_A09	Concentrative Na+-nucleoside cotransporter CNT1/CNT2
	Dj_aH_311_H05.double	Purine nucleoside phosphorylase
Amino acid transport and metabolism	Dj_CL0221_001_b2	Puromycin-sensitive aminopeptidase and related aminopeptidases
	Dj_CL2253_001_b2	Threonine/serine dehydratases
	Dj_CL1143_001_b2	Amino acid transporters
	Dj_aH_522_K10	4-aminobutyrate aminotransferase
	Dj_aH_529_E12.double	Gamma-glutamyltransferase
	Dj_CL1574_001_b2	Glycine/serine hydroxymethyltransferase
	Dj_CL0108_001_b2	Trypsin
	Dj_CL1182_001_b2	D-aspartate oxidase
	Dj_CL0627_001_b2	Dihydropteridine reductase DHPR/QDPR
	Dj_CL3658_001_b2	Kynurenine formamidase
