# Supplementary material for: Comparative transcriptome analysis between planarian Dugesia japonica and other platyhelminth species
Source: BMC Genomics. 2012 Jun 29;13:289. doi: 10.1186/1471-2164-13-289 (PMC3507646; doi:10.1186/1471-2164-13-289)
Supplement: Additional file 3 — Alignment of D. japonica and S. mediterranea PIWI2. [file 1471-2164-13-289-S3.pdf]

Additional file 2. Alignment of *D. japonica and S. mediterranea* PIWI2

D. japonica PIWI2 [EST: Dj_CL0040_001_b2]
S. mediterranea PIWI2 [Swiss-Prot: Q2Q5Y8]

Protein Alignment

Identical rate of amino acids = 93.6%
Identical and homologous substitution rate of amino acids = 98.4%

```
                         20            40            60
PIWI2_DUGJA  M-------AK LETNGSENRG R-GNRGGLRR TFRIVEPNLQ PAEIIEKTGN IGRTVKLQSN 52
PIWI2_SCHMD  MEEIPVKVAK LEANGSETRG KMGNRGGLRG TFRIVEPNLQ PADITEKTGN IGRTVKLQSN 60
                         80           100           120
PIWI2_DUGJA  FTKFSVLRSE KFMMYDSVFT QVGIPPKVKL QFIVRVCQEN RLPGAFCFDG RRLFTSEKWH 112
PIWI2_SCHMD  FTKFSVLRSE KFMMYDSVFS QVGLSPKVKL QFIVRVCQEN KLPGAFCFDG RRLFTSEKWH 120
                        140           160           180
PIWI2_DUGJA  GESDIQEFTY DDKKMTLRLV ATILPETEEY YQMINVLLNN LQIMMGQERI GKGYFLSPNI 172
PIWI2_SCHMD  GESDIQEFSH DEKKMTLRLV STILPETEEY YQMINVLLNN LQVMLGQERI GKGYFLSPNI 180
                        200           220           240
PIWI2_DUGJA  TKDEVPRNSG PTFFETNSFK VLGGFGTTLQ RGTKPTGELT TLLYIERINR VLNDNSVMKA 232
PIWI2_SCHMD  TKDEVPRNSG PTFFETNSFK VLGGFGTTLQ RGTKPTGELT TLLYIERINR VLNDNSVMKA 240
                        260           280           300
PIWI2_DUGJA  YNRRSIDQLI GRDIITKYNN KTYRISEIKE MSVDEKFEMS GRTLSYAEYF KDRYNIRLSQ 292
PIWI2_SCHMD  YNRRSIDQLI GRDIITKYNN KTYRISEIKE MNVDEKFEMG GRTLSYAEYF KERYNIRLTQ 300
                        320           340           360
PIWI2_DUGJA  GDQPFVLTRV KKPMRRERRK PGEEDKEKDK EKEKEKEKEA PQDKDMTLNI PGELCFLCGF 352
PIWI2_SCHMD  GDQPFVLTRV KKPMRRERKK KDEEGVEK-- ----EKEKEA PEEKDMTLNI PGELCFLCGF 354
                        380           400           420
PIWI2_DUGJA  SDQEKSNMDL QKSLGSVLKR EPRERLDDIP AFCNWIKSSD NANGMCNKWQ LKIENKPLEI 412
PIWI2_SCHMD  SDQEKSNMDL QKNLGCVLKR EPRERLDDIP AYCNWIKNSD AATGMCNKWQ LKIDNKPLEI 414
                        440           460           480
PIWI2_DUGJA  EGRELPPCDV ISGNNKINEK IPDDWKFGRV QFDIKRDRKH EIDVVIVDRN DFQYKNFMSD 472
PIWI2_SCHMD  EGRELPPCDV ISGGSKINEK MGDDWKFGRV QFDIKRDRKH EIDVVIVDRN DFQYKNFMND 474
                        500           520           540
PIWI2_DUGJA  VEQELRNMRI DARVGKVNTC GPNDVERCLN DAARSGSGCA KMALVFVPDD RVYAKVKSFT 532
PIWI2_SCHMD  VEQELRNMRI DARVGKVNTC GPNDVERCLN DAARSGSGCA KMALVFVPDD RVYAKVKSFT 534
                        560           580           600
PIWI2_DUGJA  MSTGLLTQCV TTRNGTNRND KRRKVVSNKT VMQIFAKFGY DPWTVEIKLR PTMIVGMDTY 592
PIWI2_SCHMD  MSTGLLTQCV TTRNGTNRND KRRKVVSSKT VMQIFAKFGY DPWTVEIKLR PTMIVGMDTY 594
                        620           640           660
PIWI2_DUGJA  HNKSSKKSIQ ASVFSINSTF TQYMSFVNQ- PKGRQEFHET LGKNFNLALE DFKKRYDILP 651
PIWI2_SCHMD  HNKSSKKSIQ ASVFSINSTF TQYMSFVVNS PKGRQEFHET LGKNFNLALE DFKKRYDILP 654
                        680           700           720
PIWI2_DUGJA  QRILVFRDGV GDNQLQFTKN FEVDAMKPLI ENIYKSNGFP VPQIIYVIVK KRVGTKLFNR 711
PIWI2_SCHMD  QRILVFRDGV GDNQLQFTKN FEVDAMKPLI ENIYKGNGFP VPQIIYVIVK KRVGTKLFNR 714
                        740           760           780
PIWI2_DUGJA  GNNPNPGTVV DKEIVKPNFY EFFLVSQRTT KGTATPTNYN VLEDTRLLTK KGTMDPMAPN 771
PIWI2_SCHMD  GNNPNPGTVV DKEIVKPNFY EFFLVSQRTT KGTATPTNYN VLEDTRLLTK KGTMDPMAPN 774
                        800           820
PIWI2_DUGJA  ELQKITYALT HLYFNWMGTI RVPVPVHYAH RLAELVGKVH KGNSPAVINE RIRNRLFYL 830
PIWI2_SCHMD  ELQKITYALT HLYFNWMGTI RVPVPVHYAH RLAELVGKVH KGSNPVAINE RIRNRLFYL 833
```

Nucleotide Alignment

Identical rate of nucleotides = 79.7%

20 40 60

PIWI2_DUGJA ATG------- ---------- ----GCTAAG TTAGAAACGA ATGGCAGTGA AAATAGAGGA 39
PIWI2_SCHMD ATGGAGGAAA TACCAGTAAA AGTAGCTAAA CTTGAAGCGA ATGGAAGTGA AACAAGAGGT 60

80 100 120

PIWI2_DUGJA AGA---GGAA ATCGCGGTGG CTTACGTAGA ACTTTCAGAA TCGTAGAGCC CAATCTCCAA 96
PIWI2_SCHMD AAAATGGGAA ATCGTGGTGG CCTGAGAGGA ACATTTAGAA TTGTTGAACC TAACTTGCAA 120

140 160 180

PIWI2_DUGJA CCGGCTGAAA TTATAGAAAA AACTGGAAAC ATAGGTAGAA CCGTCAAACT TCAATCCAAT 156
PIWI2_SCHMD CCTGCTGATA TCACTGAGAA AACAGGAAAC ATAGGAAGAA CAGTTAAATT ACAATCAAAT 180

200 220 240

PIWI2_DUGJA TTTACCAAAT TTTCTGTGTT ACGATCCGAA AAATTCATGA TGTATGATTC TGTTTTTACA 216
PIWI2_SCHMD TTTACCAAAT TTTCTGTTTT GCGTTCAGAA AAGTTCATGA TGTATGATTC CGTGTTTTCT 240

260 280 300

PIWI2_DUGJA CAAGTTGGAA TTCCACCAAA AGTCAAACTC CAATTTATTG TTAGAGTTTG CCAAGAAAAT 276
PIWI2_SCHMD CAAGTTGGTT TGTCACCCAA AGTAAAATTA CAGTTTATTG TTCGAGTCTG CCAAGAAAAT 300

320 340 360

PIWI2_DUGJA AGGTTACCTG GAGCTTTTTG TTTCGATGGA AGACGATTGT TTACAAGTGA GAAGTGGCAT 336
PIWI2_SCHMD AAATTGCCAG GGGCTTTTTG TTTTGATGGA AGACGTCTAT TTACTAGTGA AAAATGGCAT 360

380 400 420

PIWI2_DUGJA GGCGAATCTG ATATTCAAGA GTTTACCTAC GACGATAAAA AAATGACCTT ACGATTAGTT 396
PIWI2_SCHMD GGAGAATCGG ACATTCAAGA ATTTAGTCAT GATGAAAAAA AAATGACACT AAGATTGGTA 420

440 460 480

PIWI2_DUGJA GCGACCATTC TTCCAGAAAC AGAAGAATAT TATCAAATGA TAAACGTCTT ATTGAACAAC 456
PIWI2_SCHMD TCAACTATCC TTCCTGAAAC TGAAGAATAT TACCAAATGA TCAATGTATT ACTCAATAAT 480

500 520 540

PIWI2_DUGJA TTACAAATAA TGATGGGCCA AGAGCGAATT GGAAAAGGTT ACTTTTTAAG TCCCAATATA 516
PIWI2_SCHMD TTACAAGTAA TGTTAGGTCA AGAAAGAATT GGTAAAGGTT ATTTTTTAAG CCCAAATATT 540

560 580 600

PIWI2_DUGJA ACAAAAGACG AAGTTCCTCG CAATAGTGGT CCGACATTCT TTGAAACGAA TAGTTTTAAA 576
PIWI2_SCHMD ACAAAAGATG AAGTTCCCCG AAATTCTGGA CCGACATTTT TTGAAACCAA TAGTTTTAAA 600

620 640 660

PIWI2_DUGJA GTATTAGGAG GATTTGGTAC GACACTACAG AGAGGCACAA AGCCTACTGG GGAACTAACG 636
PIWI2_SCHMD GTTCTTGGGG GCTTTGGTAC AACTTTACAA AGAGGCACAA AACCAACTGG TGAATTAACC 660

680 700 720

PIWI2_DUGJA ACATTGCTCT ACATTGAGAG AATCAACAGA GTTCTAAATG ATAATTCAGT CATGAAAGCC 696
PIWI2_SCHMD ACTCTTTTAT ATATTGAGCG TATTAACAGA GTATTAAACG ATAACTCTGT TATGAAGGCA 720

740 760 780

PIWI2_DUGJA TATAATCGTC GAAGTATTGA TCAATTAATC GGACGAGACA TTATAACCAA ATACAACAAC 756
PIWI2_SCHMD TACAACAGAA GAAGTATTGA TCAGTTGATA GGCAGAGATA TCATAACAAA ATATAATAAC 780

800 820 840

PIWI2_DUGJA AAGACCTATA GAATTTCCGA AATTAAAGAA ATGAGCGTAG ATGAGAAATT TGAAATGTCA 816
PIWI2_SCHMD AAAACTTATA GGATAAGTGA AATTAAAGAA ATGAACGTAG ATGAGAAATT TGAAATGGGT 840

860 880 900

PIWI2_DUGJA GGAAGAACAT TATCTTATGC CGAATATTTT AAAGATCGAT ATAACATTCG ATTATCTCAA 876
PIWI2_SCHMD GGTAGAACTT TATCGTATGC CGAATATTTT AAGGAACGAT ATAATATTCG ATTAACTCAA 900

920 940 960

PIWI2_DUGJA GGTGACCAAC CATTTGTATT GACAAGAGTT AAAAAACCAA TGCGCCGTGA GAGAAGAAAG 936
PIWI2_SCHMD GGCGATCAAC CATTTGTTCT AACGCGAGTG AAGAAACCAA TGCGTCGAGA AAGAAAGAAA 960

980 1,000 1,020

PIWI2_DUGJA CCAGGAGAAG AAGACAAAGA AAAGGATAAA GAGAAAGAAA AAGAGAAAGA GAAAGAAGCC 996
PIWI2_SCHMD AAAGACGAAG AAGGAGTGGA AAAA------ ---------- --GAGAAAGA AAAAGAGGCT 1002

1,040 1,060 1,080

PIWI2_DUGJA CCTCAGGATA AAGATATGAC ATTGAATATT CCTGGAGAAT TGTGTTTCTT ATGTGGATTT 1056
PIWI2_SCHMD CCAGAAGAGA AGGACATGAC TCTCAATATT CCTGGAGAGT TGTGTTTCTT ATGTGGGTTT 1062

1,100 1,120 1,140

PIWI2_DUGJA TCAGATCAAG AAAAATCTAA TATGGATCTC CAAAAGAGCT TAGGAAGTGT ATTAAAAAGA 1116
PIWI2_SCHMD TCAGATCAAG AGAAATCGAA TATGGATCTG CAGAAGAATT TAGGGTGTGT TTTAAAAAGA 1122

1,160 1,180 1,200

PIWI2_DUGJA GAGCCACGTG AAAGACTTGA TGACATTCCT GCATTCTGCA ATTGGATTAA AAGTAGCGAT 1176
PIWI2_SCHMD GAGCCACGTG AAAGATTGGA TGATATACCA GCTTATTGTA ACTGGATTAA AAATAGTGAT 1182

1,220 1,240 1,260

PIWI2_DUGJA AATGCTAATG GAATGTGCAA TAAGTGGCAA TTAAAGATTG AAAATAAACC CCTCGAAATT 1236
PIWI2_SCHMD GCGGCAACTG GAATGTGCAA TAAATGGCAG CTGAAAATAG ATAACAAACC ATTAGAAATC 1242

1,280 1,300 1,320

PIWI2_DUGJA GAGGGCAGGG AGTTGCCGCC ATGTGATGTT ATTAGCGGTA ATAATAAAAT CAACGAGAAG 1296
PIWI2_SCHMD GAAGGTAGAG AATTACCTCC ATGCGATGTA ATAAGTGGTG GCAGCAAAAT TAATGAAAAA 1302

```
                                            1,340                               1,360                               1,380
                                              |                                   |                                   |
PIWI2_DUGJA  ATTCCTGATG ATTGGAAATT TGGAAGAGTT CAGTTTGATA TAAAAAGAGA CCGTAAACAT 1356
PIWI2_SCHMD  ATGGGAGATG ATTGGAAATT TGGTCGAGTT CAATTTGATA TTAAGCGTGA TCGTAAGCAT 1362
                                            1,400                               1,420                               1,440
                                              |                                   |                                   |
PIWI2_DUGJA  GAAATTGATG TAGTTATCGT CGATAGAAAT GATTTCCAGT ATAAAAATTT CATGAGTGAC 1416
PIWI2_SCHMD  GAAATCGATG TGGTCATCGT TGATCGAAAT GACTTTCAGT ATAAAAATTT TATGAATGAT 1422
                                            1,460                               1,480                               1,500
                                              |                                   |                                   |
PIWI2_DUGJA  GTAGAGCAAG AATTGAGAAA CATGCGAATT GATGCAAGAG TTGGTAAAGT AAATACATGT 1476
PIWI2_SCHMD  GTGGAACAAG AATTAAGAAA CATGAGAATT GATGCTCGAG TTGGAAAGGT TAATACTTGT 1482
                                            1,520                               1,540                               1,560
                                              |                                   |                                   |
PIWI2_DUGJA  GGTCCAAATG ACGTTGAAAG ATGTCTAAAT GATGCAGCAA GAAGTGGGAG TGGATGTGCC 1536
PIWI2_SCHMD  GGTCCAAATG ATGTTGAAAG ATGTTTGAAT GATGCCGCTA GAAGTGGAAG TGGGTGTGCT 1542
                                            1,580                               1,600                               1,620
                                              |                                   |                                   |
PIWI2_DUGJA  AAAATGGCAT TAGTTTTTGT ACCGGATGAC CGAGTTTATG CTAAAGTTAA ATCTTTCACC 1596
PIWI2_SCHMD  AAAATGGCGC TGGTATTCGT CCCAGATGAT AGAGTATATG CCAAGGTGAA ATCATTTACC 1602
                                            1,640                               1,660                               1,680
                                              |                                   |                                   |
PIWI2_DUGJA  ATGAGTACTG GTCTTTTAAC ACAATGCGTG ACAACCAGAA ATGGGACCAA CAGAAACGAT 1656
PIWI2_SCHMD  ATGAGTACTG GTCTTTTAAC CCAATGTGTA ACAACACGTA ATGGTACTAA TAGAAACGAT 1662
                                            1,700                               1,720                               1,740
                                              |                                   |                                   |
PIWI2_DUGJA  AAACGCAGAA AAGTCGTTTC CAATAAAACT GTTATGCAAA TATTTGCAAA ATTTGGATAC 1716
PIWI2_SCHMD  AAACGAAGAA AGGTGGTTTC AAGTAAAACC GTGATGCAAA TATTTGCAAA ATTCGGATAT 1722
                                            1,760                               1,780                               1,800
                                              |                                   |                                   |
PIWI2_DUGJA  GACCCATGGA CTGTTGAAAT TAAATTACGA CCAACTATGA TTGTTGGCAT GGACACCTAC 1776
PIWI2_SCHMD  GATCCATGGA CAGTTGAAAT TAAATTGAGA CCAACTATGA TTGTCGGTAT GGATACCTAT 1782
                                            1,820                               1,840                               1,860
                                              |                                   |                                   |
PIWI2_DUGJA  CATAACAAGT CTAGTAAAAA GTCAATTCAA GCATCTGTAT TCTCGATAAA CAGTACATTT 1836
PIWI2_SCHMD  CATAACAAGT CCAGCAAGAA ATCAATTCAA GCATCTGTAT TTTCGATCAA CAGCACATTT 1842
                                            1,880                               1,900                               1,920
                                              |                                   |                                   |
PIWI2_DUGJA  ACGCAATATA TGAGTTTCGT AAATCAA--- CCTAAAGGTC GACAAGAATT TCATGAAACC 1893
PIWI2_SCHMD  ACACAATATA TGAGCTTTGT TGTTAATTCT CCTAAAGGAC GTCAGGAATT CCACGAAACT 1902
                                            1,940                               1,960                               1,980
                                              |                                   |                                   |
PIWI2_DUGJA  TTAGGTAAAA ATTTTAATTT GGCTTTAGAA GATTTCAAGA AAAGATACGA TATTTTGCCG 1953
PIWI2_SCHMD  TTAGGCAAAA ATTTCAATTT AGCGTTAGAA GATTTCAAGA AACGTTATGA CATATTGCCC 1962
                                            2,000                               2,020                               2,040
                                              |                                   |                                   |
PIWI2_DUGJA  CAAAGAATTT TAGTATTTCG AGATGGTGTT GGTGACAATC AGTTGCAATT TACAAAGAAT 2013
PIWI2_SCHMD  CAGCGAATTT TAGTCTTTAG AGATGGTGTT GGAGATAACC AACTTCAATT TACTAAAAAT 2022
                                            2,060                               2,080                               2,100
                                              |                                   |                                   |
PIWI2_DUGJA  TTTGAGGTAG ATGCTATGAA ACCATTGATT GAAAATATTT ATAAAAGTAA CGGTTTCCCG 2073
PIWI2_SCHMD  TTTGAAGTTG ATGCAATGAA ACCGCTTATT GAAAATATAT ACAAAGGCAA CGGATTCCCT 2082
                                            2,120                               2,140                               2,160
                                              |                                   |                                   |
PIWI2_DUGJA  GTCCCCCAAA TTATCTACGT CATCGTTAAG AAACGAGTGG GGACTAAACT ATTCAACCGA 2133
PIWI2_SCHMD  GTTCCACAAA TCATTTATGT CATCGTAAAG AAAAGAGTCG GCACTAAGCT TTTCAACAGA 2142
                                            2,180                               2,200                               2,220
                                              |                                   |                                   |
PIWI2_DUGJA  GGCAACAATC CAAATCCAGG AACAGTTGTC GATAAAGAAA TAGTGAAACC GAACTTTTAC 2193
PIWI2_SCHMD  GGAAATAATC CAAATCCTGG AACTGTTGTG GATAAAGAAA TTGTCAAACC GAATTTCTAT 2202
                                            2,240                               2,260                               2,280
                                              |                                   |                                   |
PIWI2_DUGJA  GAGTTTTTCT TGGTTTCACA AAGAACAACG AAAGGAACAG CAACACCCAC TAATTATAAT 2253
PIWI2_SCHMD  GAATTTTTCC TAGTTTCACA AAGAACAACG AAAGGAACTG CAACTCCAAC TAATTATAAT 2262
                                            2,300                               2,320                               2,340
                                              |                                   |                                   |
PIWI2_DUGJA  GTTTTGGAAG ACACTCGATT GTTGACTAAA AAAGGAACAA TGGATCCCAT GGCTCCTAAT 2313
PIWI2_SCHMD  GTTTTAGAAG ATACTCGATT GCTAACGAAA AAAGGTACTA TGGATCCTAT GGCACCGAAT 2322
                                            2,360                               2,380                               2,400
                                              |                                   |                                   |
PIWI2_DUGJA  GAACTGCAAA AAATTACTTA CGCACTAACT CATTTATATT TCAATTGGAT GGGTACAATT 2373
PIWI2_SCHMD  GAGCTGCAAA AGATTACCTA TGCTCTAACT CATTTATATT TCAATTGGAT GGGAACTATC 2382
                                            2,420                               2,440                               2,460
                                              |                                   |                                   |
PIWI2_DUGJA  CGTGTACCTG TCCCTGTGCA TTACGCACAT CGATTGGCAG AGTTAGTGGG AAAAGTTCAC 2433
PIWI2_SCHMD  CGAGTTCCGG TTCCAGTGCA TTATGCGCAT AGATTGGCTG AATTAGTTGG AAAGGTGCAT 2442
                                            2,480                               2,500                               2,520
                                              |                                   |                                   |
PIWI2_DUGJA  AAAGGAAATA GTCCTGCTGT AATCAATGAA AGAATCAGGA ACAGGCTTTT CTATTTATAA 2493
PIWI2_SCHMD  AAGGGCTCTA ATCCCGTTGC CATTAATGAA AGAATAAGAA ATAGGCTCTT CTATTTATAA 2502
```
