# Supplementary material for: Comparative transcriptome analysis between planarian Dugesia japonica and other platyhelminth species
Source: BMC Genomics. 2012 Jun 29;13:289. doi: 10.1186/1471-2164-13-289 (PMC3507646; doi:10.1186/1471-2164-13-289)
Supplement: Additional file 4 — Alignment of D. japonica and S. mediterranea GGT1. [file 1471-2164-13-289-S4.pdf]

Additional file 3. Alignment of *D. japonica and S. mediterranea* GGT1

D. japonica GGT1 [EST: Dj_aH_529_E12.double]
S. mediterranea GGT1 [GenBank: DN304849.1]

Protein Alignment

Identical rate of amino acids = 69.8%
Identical and homologous substitution rate of amino acids = 88.3%

```
                                       20                    40                    60
                                        |                     |                     |
GGT1_DUGJA   MGNETGIIYN  DEMDDFSSPS  KANIYNLPPS  PSNYIRPYKR  PMSSMSPFIM  IDKNGQVELI  60
GGT1_SCHMD   MGTKTGIVYN  DEMDDFSSPN  KSNIYNLPPS  PSNFIMPYKR  PMSSMSPIII  VDNEGNVDLV  60
                                       80                   100                   120
                                        |                     |                     |
GGT1_DUGJA   LGASGGSRII  SSTLQVAVLA  KLFNKSIKEA  IDIPRVHHQL  LPNIITVEMN  FPDNIVKSLQ  120
GGT1_SCHMD   LGASGGSKIL  SATTQVSILS  KLLKKTIKEA  IDIPRIHHQL  IPDEVVAEMN  FPDVLVESLR  120
                                      140                   160
                                        |                     |
GGT1_DUGJA   KRGHEVQKTS  DFAVCQGIKK  SENSSF-WQC  NADFRKMGLC  DGS  162
GGT1_SCHMD   QKGHIVRKDP  DYAVCQGIMK  SLDSAYSWQC  NADFRKMGLC  DGF  163
```

Nucleotide Alignment

Identical rate of nucleotides = 72.8%

```
                                           20                    40                    60
                                            |                     |                     |
GGT1_DUGJA-1   ATGGGAAATG  AAACTGGAAT  CATTTACAAT  GATGAAATGG  ATGACTTTTC  CTCACCAAGT  60
GGT1_SCHMD-1   ATGGGAACCA  AAACTGGAAT  AGTTTACAAC  GACGAGATGG  ACGATTTCTC  TTCTCCGAAC  60
                                           80                   100                   120
                                            |                     |                     |
GGT1_DUGJA-1   AAAGCAAATA  TTTATAATCT  ACCGCCTTCT  CCAAGTAACT  ATATAAGACC  GTATAAGCGA  120
GGT1_SCHMD-1   AAATCAAACA  TTTACAACTT  ACCACCATCT  CCCAGTAACT  TCATTATGCC  ATATAAAAGA  120
                                          140                   160                   180
                                            |                     |                     |
GGT1_DUGJA-1   CCGATGTCGT  CTATGTCTCC  ATTTATTATG  ATCGATAAAA  ATGGACAAGT  AGAATTAATT  180
GGT1_SCHMD-1   CCAATGTCAT  CCATGTCTCC  GATTATCATT  GTTGATAATG  AAGGAAATGT  TGATTTAGTT  180
                                          200                   220                   240
                                            |                     |                     |
GGT1_DUGJA-1   CTTGGTGCTT  CGGGAGGCAG  TAGAATCATT  TCAAGCACAT  TACAAGTTGC  AGTACTTGCT  240
GGT1_SCHMD-1   CTTGGAGCTT  CTGGAGGCAG  TAAAATACTT  TCAGCTACAA  CACAAGTTTC  TATTCTTTCC  240
                                          260                   280                   300
                                            |                     |                     |
GGT1_DUGJA-1   AAATTGTTCA  ATAAAAGCAT  AAAAGAAGCG  ATTGACATTC  CACGAGTTCA  TCATCAATTG  300
GGT1_SCHMD-1   AAATTGTTGA  AAAAGACCAT  TAAAGAGGCA  ATAGACATTC  CCCGAATTCA  CCATCAATTA  300
                                          320                   340                   360
                                            |                     |                     |
GGT1_DUGJA-1   TTGCCTAATA  TAATCACTGT  TGAAATGAAC  TTTCCTGATA  ATATTGTTAA  AAGTTTGCAG  360
GGT1_SCHMD-1   ATTCCAGATG  AAGTTGTTGC  GGAAATGAAT  TTTCCGGATG  TTCTAGTTGA  AAGTCTGAGG  360
                                          380                   400                   420
                                            |                     |                     |
GGT1_DUGJA-1   AAAAGAGGTC  ATGAAGTACA  AAAGACGTCT  GATTTTGCGG  TTTGTCAAGG  TATCAAAAAA  420
GGT1_SCHMD-1   CAAAAAGGTC  ATATTGTGAG  AAAAGATCCT  GATTATGCAG  TATGTCAAGG  CATAATGAAA  420
                                          440                   460                   480
                                            |                     |                     |
GGT1_DUGJA-1   TCAGAAAATT  CGTC---TTT  CTGGCAATGT  AATGCTGACT  TTAGAAAAAT  GGGACTATGT  477
GGT1_SCHMD-1   TCTTTGGATT  CAGCATATTC  ATGGCAGTGC  AATGCTGACT  TTAGGAAAAT  GGGGTTATGT  480

GGT1_DUGJA-1   GATGGTTCAT  AA  489
GGT1_SCHMD-1   GATGGATTTT  GA  492
```
